# Unrelated donor versus matched sibling donor in adults with acute myeloid leukemia in first relapse: an ALWP-EBMT study

**DOI:** 10.1186/s13045-016-0321-y

**Published:** 2016-09-17

**Authors:** Annalisa Ruggeri, Giorgia Battipaglia, Myriam Labopin, Gerhard Ehninger, Dietrich Beelen, Johanna Tischer, Arnold Ganser, Rainer Schwerdtfeger, Bertram Glass, Jurgen Finke, Mauricette Michallet, Matthias Stelljes, Pavel Jindra, Renate Arnold, Nicolaus Kröger, Mohamad Mohty, Arnon Nagler

**Affiliations:** 1 Service d’Hématologie et Thérapie Cellulaire, Hôpital Saint Antoine, AP-HP, 184 Rue du Faubourg Saint Antoine, 75012 Paris, France; 2 Department of Hematology and Marrow Transplantation, University Federico II of Naples, Naples, Italy; 3 Medical Clinic and Polyclinic, University Hospital Carl Gustav Carus, Technical University Dresden, Dresden, Germany; 4 Department of Bone Marrow Transplantation, University Hospital Essen, Essen, Germany; 5 Department of Internal Medicine III, UH of Munich (LMU), Munich, Germany; 6 Department of Hematology, Hemostasis, Oncology, and Stem Cell Transplantation, Hannover Medical School, Hannover, Germany; 7 Department of Haematology, Oncology Helios-Klinikum Berlin-Buch, Schwanebecker Chaussee 50, 13125 Berlin, Germany; 8 Department of Hematology, Oncology and Stem Cell Transplantation, Asklepios Hospital St. Georg, Hamburg, Germany; 9 Department of Hematology/Oncology and Stem Cell Transplantation, University Medical Center, Freiburg, Germany; 10 Department of Hematology, Lyon-Sud Hospital, Hospices Civils de Lyon, Pierre Bénite, France; 11 Department of Medicine A, University of Muenster, Munster, Germany; 12 Departments of Hematology/Oncology, Charles University Hospital, Pilsen, Czech Republic; 13 Charité Universitatsmedizin, 10117 Berlin, Germany; 14 University Medical Center Hamburg-Eppendorf, Hamburg, Germany; 15 Hôpital Saint-Antoine, Paris University UPMC, INSERM U938, Paris, France; 16 Université Pierre and Marie Curie, Paris, France; 17 Hematology Division, Chaim Sheba Medical Center, Tel Hashomer, Israel; 18 ALWP Office, Hôpital Saint Antoine, AP-HP, Paris, France

**Keywords:** Acute myeloid leukemia, Relapse, Matched sibling donor, Unrelated donor

## Abstract

**Background:**

Allogeneic stem cell transplantation is the only curative option for patients with acute myeloid leukemia (AML) experiencing relapse. Either matched sibling donor (MSD) or unrelated donor (UD) is indicated.

**Methods:**

We analyzed 1554 adults with AML transplanted from MSD (*n* = 961) or UD (*n* = 593, HLA-matched 10/10, *n* = 481; 9/10, *n* = 112). Compared to MSD, UD recipients were older (49 vs 52 years, *p* = 0.001), transplanted more recently (2009 vs 2006, *p* = 0.001), and with a longer interval to transplant (10 vs 9 months, *p* = 0.001). Conditioning regimen was more frequently myeloablative for patients transplanted with a MSD (61 vs 46 %, *p* = 0.001). Median follow-up was 28 (range 3–157) months.

**Results:**

Cumulative incidence (CI) of neutrophil engraftment (*p* = 0.07), grades II–IV acute GVHD (*p* = 0.11), chronic GVHD (*p* = 0.9), and non-relapse mortality (NRM, *p* = 0.24) was not different according to the type of donor. At 2 years, CI of relapse (relapse incidence (RI)) was 57 vs 49 % (*p* = 0.001). Leukemia-free survival (LFS) at 2 years was 21 vs 26 % (*p* = 0.001), and overall survival (OS) was 26 vs 33 % (*p* = 0.004) for MSD vs UD, respectively. Chronic GVHD as time-dependent variable was associated with lower RI (HR 0.78, *p* = 0.05), higher NRM (HR 1.71, *p* = 0.001), and higher OS (HR 0.69, *p* = 0.001). According to HLA match, RI was 57 vs 50 vs 45 %, (*p* = 0.001) NRM was 23 vs 23 vs 29 % (*p* = 0.26), and LFS at 2 years was 21 vs 27 vs 25 % (*p* = 0.003) for MSD, 10/10, and 9/10 UD, respectively. In multivariate analysis adjusted for differences between the two groups, UD was associated with lower RI (HR 0.76, *p* = 0.001) and higher LFS (HR 0.83, *p* = 0.001) compared to MSD. Interval between diagnosis and transplant was the other factor associated with better outcomes (RI (HR 0.62, *p* < 0.001) and LFS (HR 0.67, *p* < 0.001)).

**Conclusions:**

Transplantation using UD was associated with better LFS and lower RI compared to MSD for high-risk patients with AML transplanted in first relapse.

## Background

Although the outcome of patients diagnosed with acute myeloid leukemia (AML) has improved, disease recurrence remains the leading cause of treatment failure, with only a minority of patients who durably benefit from current reinduction therapies. For patients achieving complete remission (CR) after salvage chemotherapy, the overall relapse risk is nearly 45–50 %, but this is highly variable and is primarily determined by the biology of the disease [1]. For patients with AML, allogeneic hematopoietic stem cell transplantation (HSCT) is a curative option. Recent improvements in donor availability, conditioning regimens, and supportive care broadened the applicability of HSCT in nearly all patients with AML with better long-term outcomes. Moreover, the use of post-transplant immunomodulating strategies allowed long-term survival and better disease control.

Both myeloablative (MAC) and reduced-intensity (RIC) conditioning regimens are suitable options for treating these patients allowing disease control. In the MAC setting, regimens containing either busulfan or total body irradiation (TBI) are effective for refractory AML with a reported 2-year leukemia-free survival (LFS) of less than 30 % with both regimens [2]. On the other hand, the introduction of RIC made HSCT feasible even in elderly or in young patients with significant comorbidities, not eligible for standard MAC [3]. However, post-transplant relapse remains a challenge in RIC, especially in high-risk patients with AML in primary relapse.

In this setting, donor search might be started as soon as possible. For patients lacking a full-matched sibling donor (MSD), 35–40 % may have an unrelated donor (UD) available in donor registries [4]. Prior studies reported comparable results of HSCT from MSD versus UD [5]; however, up to date, no studies investigated the outcomes of patients transplanted from either a MSD or UD for AML in first relapse.

Understanding the impact of donor source in patients with active disease represents an area of active investigation and might be critical in the therapeutic decision-making process. With this background, we analyzed patients with AML in first relapse reported to the European Society for Blood and Marrow Transplantation (EBMT) registry from 2000 to 2012.

## Methods

### Study design and definition

We retrospectively analyzed adult patients (≥18 years) diagnosed with AML who underwent their first allogeneic HSCT in primary relapse between 2000 and 2012, using either a MSD or matched 10/10 or mismatched 9/10 UD. Patients transplanted using cord blood or haploidentical donors were excluded to homogenously compare adult full-matched sibling or unrelated donors. The EBMT registry is a voluntary working group of more than 500 transplant centers, mostly located in Europe, that are required to report all consecutive HSCT and follow-up data once a year. Audits are routinely undertaken to establish the accuracy of the data.

Karyotype abnormalities were classified as favorable, intermediate or high risk as previously described [6]. Conditioning regimen was MAC in case of administration of total body irradiation (TBI) at a dose greater than 6 Gray (Gy), oral busulfan greater than 8 mg/kg, or intravenous busulfan greater than 6.4 mg/kg. All patients provided informed consent for transplant according to the declaration of Helsinki. The Review Board of the EBMT approved this study.

### Definitions and end points of the study

The primary endpoint of the study was LFS according to the type of donor (MSD vs UD). Secondary endpoints included overall survival (OS), neutrophil engraftment, acute and chronic graft versus host disease (GVHD), non-relapse mortality (NRM), and relapse incidence (RI). LFS was calculated from the date of transplant until relapse or last disease-free follow-up. Neutrophil engraftment was defined as achieving absolute neutrophil count ≥0.5 × 10^9^/l for three consecutive days. The diagnosis and grading of acute and chronic GVHD was performed using the standard criteria [7]. Relapse and death from any cause were considered events. NRM was defined as death without prior relapse.

### Statistical analyses

Median values and ranges were used for continuous variables and percentages for categorical variables. Patient-, disease-, and transplant-related variables of the groups were compared using Chi-square or Fischer exact test for categorical variables, and Mann–Whitney test for continuous variables. The probabilities of OS and LFS were calculated using the Kaplan–Meier [8] method and the log-rank test for univariate comparisons.

Neutrophil engraftment, grades II–IV acute and chronic GVHD, relapse, and NRM were calculated by using the cumulative incidence (CI) estimator to accommodate competing risks. For NRM, relapse was the competing risk, and for relapse, the competing risk was NRM.

Multivariate analyses adjusted for differences between the groups were performed using Cox proportional hazards regression model for LFS and OS, and Fine and Gray’s [9] proportional hazards regression model for engraftment, GVHD, NRM, and relapse. Chronic GVHD was analyzed as time-dependent factor using Cox model for LFS and OS. *p* values were two-sided. Statistical analyses were performed with the SPSS 19 (SPSS Inc./IBM, Armonk, NY, USA) and R 3.0 (R Development Core Team, Vienna, Austria) software packages.

## Results

### Patient and transplant characteristics

Baseline characteristics are summarized in Table 1. Briefly, 1554 patients fulfilling the inclusion criteria were identified; of these, 961 were transplanted with a MSD and 481 with an UD (HLA-matched 10/10, *n* = 481, 9/10, *n* = 112). There were some differences between the two groups: UD recipients were older (52 vs 49 years; *p* < 0.001) and they underwent HSCT more recently (2006 vs 2009 for UD recipients; *p* < 0.001) compared to MSD recipients. Time from diagnosis to HSCT was longer for UD recipients (10 vs 8 months, *p* < 0.001). A slightly higher proportion of secondary AML was observed in the MSD group (125 vs 113 in the UD group, *p* < 0.003).

**Table 1:** Patient, disease, and transplant characteristics

Characteristic (%)	MSD (*n* = 961)	UD (*n* = 593)	*p* value
Median follow-up, months	30 (1–157)	27 (1–137)	0.28
Median age, years (range)	49 (18–75)	52 (18–77)	<0.01
Median year of HSCT (range)	2006 (2000–2012)	2009 (2000–2012)	<0.01
Interval from diagnosis to HSCT, months (range)	8.6 (2–155)	10.3 (2.5–186)	<0.01
De novo AML	836 (87)	480 (81)	<0.01
sAML	125 (13)	113 (19)	
Prior auto-HSCT	57 (6)	41 (7)	0.59
Donor’s gender			
Male	500 (52)	417 (72)	<0.01
Female	452 (48)	162 (28)	
CMV positive serostatus			<0.01
Patient	472 (69)	210 (37)	
Donor	417 (62)	244 (42)	
Conditioning regimen			
MAC	559 (60)	269 (46)	<0.01
RIC	365 (40)	316 (54)	
Conditioning details			
BuCy	155 (16)	81 (14)	<0.01
BuFlu	135 (14)	112 (19)	
FluMel	89 (9)	81 (14)	
Threo-based	36 (4)	26 (4)	
MAC TBI	178 (1)	79 (13)	
RIC TBI	32 (3)	13 (2)	
Other	336 (35)	201 (34)	
Stem cell source			
BM	107 (11)	60 (10)	0.14
PBSC	854 (89)	533 (90)	
GVHD prophylaxis			
CsA alone	115 (12)	81 (14)	<0.01
CsA + MMF	182 (19)	187 (31)	
CsA + MTX	362 (38)	246 (41)	
Other	68 (7)	75 (13)	
Missing	234 (24)	4 (1)	
In vivo TCD			<0.01
ATG	167 (17)	388 (65)	
Alemtuzumab	20 (2)	47 (8)	
No	521 (54)	154 (26)	
Missing	235 (24)	4 (1)	

*Abbreviations*: *MSD* matched sibling donor, *UD* unrelated donor, *HSCT* hematopoietic stem cell transplantation, *sAML* secondary acute myeloid leukemia, *auto-HSCT* autologous stem cell transplantation, *CMV* cytomegalovirus, *MAC* myeloablative conditioning regimen, *RIC* reduced-intensity conditioning regimen, *Bu* busulfan, *Cy* cyclophosphamide, *Flu* fludarabine, *Mel* melphalan, *Threo* threosulfan, *TBI* total body irradiation, *BM* bone marrow, *PBSC* peripheral blood stem cells, *CsA* cyclosporine A, *MMF* mycophenolate mophetil, *MTX* methotrexate, *TCD* T-cell depletion, *ATG* antithymocyte globulin

The majority of the patients underwent a MAC regimen, especially in the MSD group (60 vs 46 % for the UD group, *p* < 0.001); 186 patients received a combination of a short intensive course of chemotherapy, followed by a RIC regimen (FLAMSA-regimen) as previously described [10], including 125 TBI-containing and 66 busulfan-containing regimen. In vivo T cell depletion was more frequently used in UD group (99 vs 75 %, *p* < 0.001). Ninety percent of patients in both groups (*p* = 0.14) received peripheral blood cells (PBSC) as stem cell source.

### Engraftment, acute, and chronic GVHD

The CI of neutrophil engraftment was 93 vs 92 % in MSD and UD recipients, respectively (*p* = 0.07). The median time for neutrophil engraftment was of 15 days (range 6–90), with no difference between the groups. Graft failure occurred in nearly 5 % of patients in each group (*p* = 0.73).

According to the type of donor CI of day-100 grades II–IV acute GVHD was 26 vs 30 % (*p* = 0.11), in MSD and UD, respectively. There was no difference in the CI of chronic GVHD at 2 years in the 2 groups (25 %, *p* = 0.90).

### Relapse and NRM

Within 100 days after HSCT, 72 % (*n* = 403) of UD recipients achieved CR, versus 66 % (*n* = 586) in MSD group (*p* = 0.02).

**Fig. 1:**
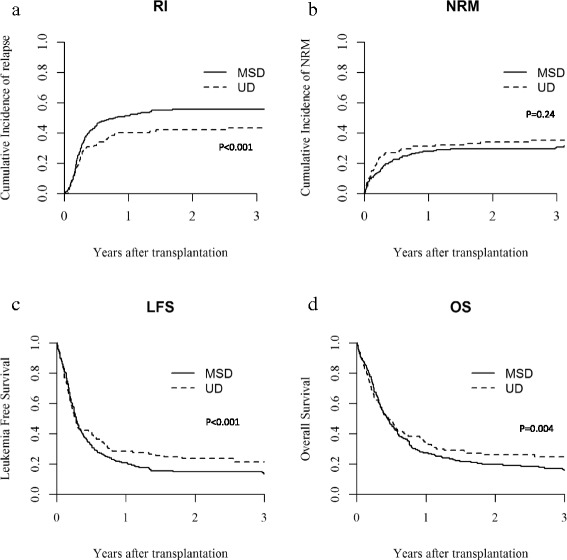
**a** Relapse incidence. **b** Non-relapse mortality. **c** Leukemia-free survival. **d** Overall survival by type of donor

At 2 years, CI of relapse was higher in MSD recipients (57 vs 49 % in UD recipients, *p* < 0.001), while NRM was similar in the two groups (23 % in MSD vs 24 % in UD, *p* = 0.24) (Fig. 1a, b).

In multivariate analysis (Table 2), patients transplanted with UD (HR = 0.76, 95 % CI 0.64–0.89; *p* < 0.001), and those with a longer interval between diagnosis and HSCT (HR = 0.62, 95 % CI 0.53–0.72; *p* < 0.001) showed lower relapse. RIC regimen was independently associated with higher relapse (HR = 1.21, 95 % CI 1.02–1.42; *p* < 0.03). Also, chronic GVHD as time-dependent variable was independently associated with a lower RI (HR = 0.78, 95 % CI 0.61–1.00; *p* < 0.05) and a higher NRM (HR = 1.71, 95 % CI 1.18–2.48; *p* < 0.001). Causes of death are reported in Table 3.

**Table 2:** Multivariate analysis for patients with AML in primary relapse

Variable	HR (95 % CI)	*p*
RI		
UD vs MSD	0.76 (0.64–0.89)	<0.01
Age^a^	0.99 (0.99–1.00)	0.02
Time from dx to Tx >9 months	0.62 (0.53–0.72)	<0.01
Female donor to male recipient	0.91 (0.74–1.11)	0.35
sAML	0.99 (0.79–1.23)	0.92
previous auto-HSCT	1.22 (0.86–1.73)	0.26
Positive recipient CMV serology	0.96 (0.80–1.15)	0.68
Positive donor CMV serology	1.03 (0.86–1.22)	0.77
RIC	1.21 (1.02–1.42)	0.03
NRM		
UD vs MSD	1.04 (0.80–1.35)	0.79
Age^a^	1.02 (1.01–1.03)	<0.01
Time from dx to Tx >9 months	0.82 (0.64–1.06)	0.12
Female donor to male recipient	0.86 (0.62–1.21)	0.40
sAML	1.39 (1.03–1.87)	0.03
previous auto-HSCT	1.49 (0.91–2.43)	0.11
Positive recipient CMV serology	0.98 (0.74–1.30)	0.89
Positive donor CMV serology	0.98 (0.74–1.28)	0.86
RIC	0.96 (0.74–1.24)	0.74
LFS		
UD vs MSD	0.83 (0.72–0.96)	0.01
Age^a^	1.00 (0.99–1.00)	0.82
Time from dx to Tx >9 months	0.67 (0.59–0.77)	<0.01
Female donor to male recipient	0.90 (0.75–1.07)	0.22
sAML	1.11 (0.93–1.32)	0.26
previous auto-HSCT	1.29 (0.97–1.71)	0.08
Positive recipient CMV serology	0.97 (0.84–1.13)	0.72
Positive donor CMV serology	1.01 (0.87–1.17)	0.87
RIC	1.13 (0.98–1.30)	0.09
OS		
UD vs MSD	0.89 (0.77–1.03)	0.12
Age^a^	1.00 (1.00–1.01)	0.36
Time from dx to Tx >9 months	0.71 (0.61–0.81)	<0.01
Female donor to male recipient	0.86 (0.72–1.03)	0.10
sAML	1.09 (0.91–1.31)	0.34
previous auto-HSCT	1.25 (0.93–1.68)	0.15
Positive recipient CMV serology	0.96 (0.82–1.12)	0.61
Positive donor CMV serology	0.99 (0.85–1.16)	0.92
RIC	1.01 (0.88–1.17)	0.88

*Abbreviations*: *HR* hazard ratio, *CI* confidence interval, *RI* relapse incidence, *NRM* non-relapse mortality, *LFS* leukemiafree survival, *OS* overall survival, *UD* unrelated donor, *MSD* matched sibling donor, *cont* continuous, *dx* diagnosis, tx transplant, *sAML* secondary acute myeloid leukemia, *auto-HSCT* autologous hematopoietic stem cell transplantation, *CMV* cytomegalovirus, *RIC* reduced-intensity conditioning regimen

^a^As continuous variable

**Table 3:** Causes of death for patients with MSD and UD

	MSD	UD
Cardiac toxicity	3 (0.4 %)	3 (0.8 %)
Hemorrhage	12 (1.7 %)	5 (1.3 %)
Failure/Rejection	3 (0.4 %)	1 (0.3 %)
Veno Occlusive Disease	11 (1.6 %)	14 (3.7 %)
Infection	105 (15.1 %)	64 (16.8 %)
Interstitial Pneumonia	11 (1.6 %)	7 (1.8 %)
Graft versus host disease	72 (10.4 %)	50 (13.1 %)
Original disease	436 (62.3 %)	222 (58.1 %)
Secondary malignancy	2 (0.3 %)	0 (0 %)
Other transplant related	32 (4.6 %)	9 (2.4 %)
Multi Organ Failure	8 (1.2 %)	7 (1.8 %)
Unknown	14	5

### LFS and OS

With a median follow-up of 2.4 (range 0.3–13) years, the 2 years probability of LFS was 21 vs 26 % (*p* < 0.001) and OS was 26 vs 33 % (*p* < 0.004) for MSD and UD recipients, respectively (Fig. 1c, d). In adjusted multivariate analysis (Table 2), LFS was higher for patients transplanted with UD (HR = 0.83, 95 % CI 0.72–0.96; *p* < 0.01) and for those with a shorter interval between diagnosis and HSCT (HR = 0.67, 95 % CI 0.59–0.77; *p* < 0.001). Chronic GVHD as time-dependent variable was associated with higher OS (HR = 0.69, 95 % CI 0.56–0.84; *p* < 0.001), and no differences were observed for LFS (HR = 0.99, 95 % CI 0.80–1.21; *p* = 0.90).

### Outcomes according to HLA-match

We performed a subgroup analysis to evaluate outcomes according to HLA disparity (UD 10/10 or 9/10 as compared to MSD). There were no differences for neutrophil engraftment (92 vs 89 vs 93 %, *p* = 0.20), chronic GVHD (25 % in all groups, *p* = 0.96), and NRM (23 vs 23 vs 30 %, *p* = 0.26) between the three groups. CI of relapse was higher for MSD as compared to 10/10 or 9/10 UD (57 vs 50 vs 45 %, *p* = 0.0003). Patients in the MSD group had lower LFS (20 % in MSD vs 27 % in 10/10 and 25 % in 9/10 UD, *p* = 0.003), and lower OS (26 % in MSD vs 34 % in 10/10 and 29 % in 9/10 UD, *p* = 0.01). No differences were found in the multivariate analysis (Table 4) for MSD versus 9/10 UD for RI (HR = 0.77, 95 % CI 0.57–1.05; *p* = 0.10), NRM (HR = 1.32, 95 % CI 0.87–2.00; *p* = 0.19), LFS (HR = 0.92, 95 % CI 0.72–1.18; *p* = 0.53), and OS (HR = 1.00, 95 % CI 0.78–1.29; *p* = 0.99).

**Table 4:** Multivariate analysis for patients with AML in primary relapse according to HLA mismatch

Variable	HR (95 % CI)	*p*
RI		
UD 10/10	0.75 (0.63–0.90)	<0.01
UD 9/10	0.77 (0.57–1.05)	0.10
Age^a^	0.99 (0.99–1.00)	0.02
Time from dx to Tx >9 mo	0.62 (0.52–0.72)	<0.01
Female donor to male recipient	0.91 (0.74–1.11)	0.34
sAML	0.99 (0.79–1.23)	0.92
previous auto-HSCT	1.22 (0.86–1.73)	0.26
Positive recipient CMV serology	0.96 (0.80–1.15)	0.68
Positive donor CMV serology	1.03 (0.86–1.22)	0.77
RIC	1.21 (1.02–1.42)	0.03
NRM		
UD 10/10	0.97 (0.74–1.29)	0.86
UD 9/10	1.32 (0.87–2.00)	0.19
Age^a^	1.02 (1.01–1.03)	<0.01
Time from dx to Tx >9 mo	0.81 (0.62–1.04)	0.10
Female donor to male recipient	0.86 (0.61–1.20)	0.38
sAML	1.40 (1.04–1.90)	0.03
previous auto-HSCT	1.50 (0.92–2.45)	0.10
Positive recipient CMV serology	0.96 (0.72–1.28)	0.80
Positive donor CMV serology	0.98 (0.75–1.29)	0.90
RIC	0.96 (0.74–1.25)	0.78
LFS		
UD 10/10	0.81 (0.70–0.94)	0.01
UD 9/10	0.92 (0.72–1.18)	0.53
Age^a^	1.00 (0.99–1.00)	0.82
Time from dx to Tx >9 mo	0.67 (0.58–0.76)	<0.01
Female donor to male recipient	0.89 (0.75–1.07)	0.21
sAML	1.11 (0.93–1.33)	0.24
previous auto-HSCT	1.30 (0.98–1.72)	0.07
Positive recipient CMV serology	0.97 (0.83–1.13)	0.67
Positive donor CMV serology	1.01 (0.88–1.17)	0.86
RIC	1.13 (0.98–1.30)	0.08
OS		
UD 10/10	0.87 (0.74–1.01)	0.07
UD 9/10	1.00 (0.78–1.29)	0.99
Age^a^	1.00 (1.00–1.01)	0.36
Time from dx to Tx >9 mo	0.70 (0.61–0.81)	<0.01
Female donor to male recipient	0.85 (0.71–1.03)	0.09
sAML	1.10 (0.91–1.32)	0.31
previous auto-HSCT	1.25 (0.93–1.69)	0.14
Positive recipient CMV serology	0.95 (0.81–1.12)	0.56
Positive donor CMV serology	0.99 (0.85–1.16)	0.94
RIC	1.01 (0.88–1.17)	0.86

*Abbreviations*: *HR* hazard ratio, *CI* confidence interval, *RI* relapse incidence, *NRM* non-relapse mortality, *LFS* leukemia-free survival, *OS* overall survival, *UD* unrelated donor, *MSD* matched sibling donor, *cont* continuous, *dx* diagnosis, *tx* transplant, *sAML* secondary acute myeloid leukemia, *auto-HSCT* autologous hematopoietic stem cell transplantation, *CMV* cytomegalovirus, *RIC* reduced-intensity conditioning regimen

^a^As continuous variable

## Discussion

AML recurrence still represents one of the most difficult scenarios associated with poor prognosis. Most patients often do not respond to reinduction therapies which are also hampered by toxicities. This might leave patients unfit for additional therapies. On the other hand, HSCT is a treatment strategy that may offer possibility of cure, although survival does not exceed 20 to 35 % at 4 years [11, 12]. The increased availability of donors, the use of non-myeloablative or RIC regimens, and the progress in supportive care, translating in survival improvements, made HSCT available and possible in almost all patients. Therefore, a rapid donor search should be promptly initiated in high-risk AML patients [4].

In the present study, we compared the outcomes of patients undergoing allogeneic HSCT for AML in primary relapse either from a MSD or from a matched or single-allele mismatched UD.

Several retrospective studies on patients transplanted with active disease have been reported. The French Society of Bone Marrow Transplantation (SFGM) reported outcomes of 379 patients transplanted for high-risk AML with a 5-year probability of LFS approximately of 10 %. In multivariate analysis the use of a matched or mismatched UD was associated with lower OS [13].

Few retrospective studies or case series limited to a small subset of highly selected patients receiving a MAC HSCT for active AML have been reported in the literature [13, 14]: a large CIBMTR study in AML in relapse or primary induction failure showed a 3-year OS of 19 %.

The German Transplant Study in 113 patients undergoing a RIC transplant showed a probability of LFS of 49 % for patients with less than 5 % bone marrow blasts and 14 % for those with more than 20 % marrow blasts at time of HSCT [15]. Moreover, patients transplanted from a MSD had better event-free survival as compared to patients transplanted from UD. A phase II study from van Besien et al. in patients with AML or myelodysplastic syndrome included 28 patients with active AML undergoing a RIC transplant with fludarabine, melphalan, and alemtuzumab, with a 1-year LFS of 25 %. No differences were found in the whole population outcomes according to the type of donor [16].

In our cohort, we observed that patients undergoing a RIC had a higher risk of relapse when compared to MAC transplant, in accordance with previous reports [17, 18].

Some of the patients in the current study received a sequential regimen: however, numbers were too small to analyze the impact of this regimen on HSCT outcomes.

Compared to MSD recipients, in our series patients transplanted with UD experienced higher OS, LFS, and lower RI. This was confirmed in the multivariate analysis, where the type of donor was an independent risk factor for LFS and relapse incidence. This could be related to a stronger graft versus leukemia (GVL) effect in the UD group due to a higher likelihood of mismatches in minor histocompatibility antigens [19].

Increase in GVHD in case of UD is reported both in the MAC and RIC setting, with a correlation between severity of GVHD and HLA mismatch (29). Importantly in our series, acute and chronic GVHD were not different either according to the type of donor or to HLA mismatches. However, given the retrospective nature of our study, we were not able to evaluate the effect of the single-allele mismatch and the HLA locus for addressing the GVL effect.

In the UD setting, a lower incidence of relapse in patients experiencing chronic GVHD has been reported [20]. Of note, in our series, in accordance with previous reports [21], chronic GVHD as a time-dependent variable was associated with lower risk of RI and higher OS.

Similar results were found by Lee et al. who reported a better disease control in extensive as compared to limited chronic GVHD, this being counterbalanced by morbidity from chronic GVHD [22].

When focusing on conditioning intensity, Weisdorf et al. reported a longer GVL benefit in patients experiencing GVHD after a RIC transplant as compared to patients undergoing a MAC transplant [23]. This study highlights the importance of immune modulation, especially in the context of RIC transplant.

Importantly, improvements in supportive care after HSCT over the years, including the advent of new antifungal and antiviral drugs, allowed better survival and reduction of NRM, with a wider number of patients eligible for post-transplant immune modulation with better disease control.

One may argue that patients receiving RIC may have benefit of earlier withdrawal of immunosuppressive treatment or post HSCT maintenance therapy, or donor lymphocyte infusion and therefore without differences in LFS. However, we did not investigate the type of post HSCT strategy, being related to the different transplant policy.

We confirm that the interval from diagnosis to HSCT was one of the factors associated with relapse, LFS, and OS, independently from the type of donor, highlighting the need to rapidly look for a donor and perform HSCT early to ensure long-term disease control. The place of unmanipulated haploidentical donor transplant need to be considered in this setting to establish the adapted algorithm for donor selection in this setting of patients.

Our study has some limitations as a retrospective registry analysis. Although patients were categorized by their disease biology and the multivariate modeling may have adjusted for various patient-, disease-, and transplantation-related factors, there may be factors of importance that we have not been able to take into account, such as the reason why those patients were not transplanted in first CR.

Further studies are needed to highlight the place of sequential approach in such a high-risk patients with otherwise limited options, as reported also using other regimen combination [24], and other post-transplant strategies such as early withdrawal of immunosuppressive treatment and immunomodulating agents or preemptive use of donor lymphocyte infusions should be considered early in this high-risk population to enhance long-term disease control.

## Conclusions

Our data suggest that the use of UD is feasible and effective in patients with AML undergoing allogeneic HSCT in primary relapse, with even better outcomes in terms of LFS, RI and OS in patients transplanted from MSD. The role of one antigen mismatch UD in this setting should be evaluated in a larger and more homogenous series. Moreover, as timing to HSCT still remains one of the most important factors influencing transplant outcomes, it is important to promptly start donor search on the registries when a MSD is not available, in order to identify a suitable UD. Our data confirm that a strong disease control is possible with a single-allele mismatched UD with no increased toxicity. In patients not eligible for a full intensity regimen, use of RIC regimens, might be a valid option to improve disease control and survival. Despite better results for patients given UD, outcome of patients transplanted in primary relapse is still not satisfactory. Further prospective studies investigating the implementation of post-transplant immunomodulating strategies are warranted in this high-risk subset of patients.
